# Supplementary material for: A semi-automated technique for adenoma quantification in the ApcMin mouse using FeatureCounter
Source: Sci Rep. 2020 Feb 20;10:3064. doi: 10.1038/s41598-020-60020-7 (PMC7033248; doi:10.1038/s41598-020-60020-7)
Supplement: Supplementary file 1 — Supplementary Info. [file 41598_2020_60020_MOESM1_ESM.pdf]

A semi-automated technique for adenoma quantification in the *Apc*^{Min} mouse using *FeatureCounter*

Amy L. Shepherd[1], A. Alexander T. Smith[1], Kirsty A. Wakelin[1], Sabine Kuhn, Jianping Yang, David A. Eccles[1] and Franca Ronchese[1*]

[1]Malaghan Institute of Medical Research, Wellington, New Zealand.

Supplementary Information

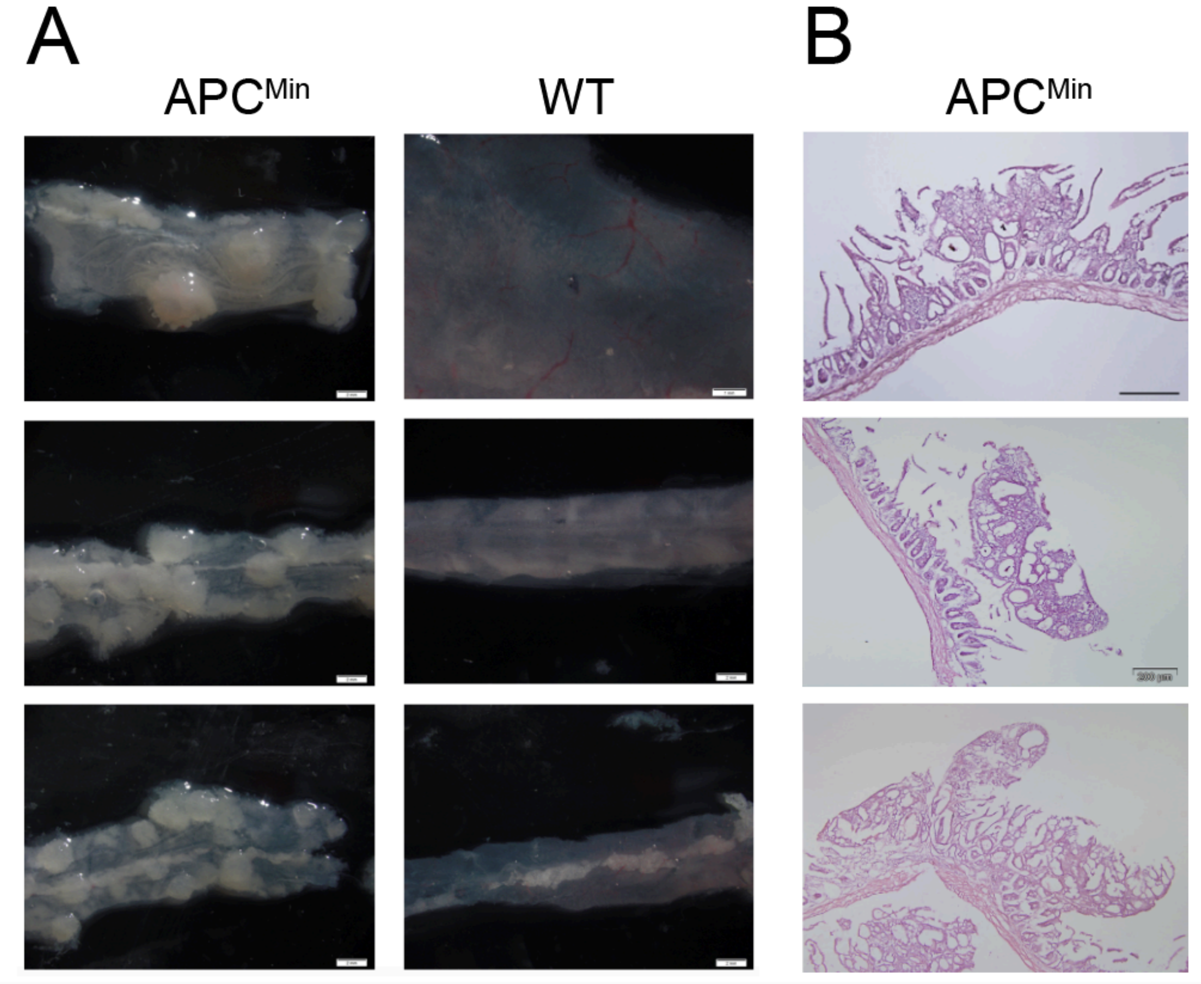

Supplementary Figure 1. Intestinal tumours in APC/Min mice and WT littermates.
APC/Min mice were sacrificed at the first sign of anemia; WT littermates were sacrificed at the same time. Intestinal tissue was collected for processing and tumour visualization as indicated.
(**A**) Intestinal tissue was opened longitudinally, washed with PBS, then immediately examined at low magnification under a stereomicroscope. Images show Duodenum (upper row), Upper jejunum (middle row) and Ileum (lower row). Size bars correspond to 1 mm (WT duodenum) or 2 mm (all other images).
(**B**) Intestinal tissue was collected and examined macroscopically. Areas corresponding to putative tumours were removed, fixed in formalin, paraffin embedded, sectioned and stained with H&E, and examined microscopically. Size bars correspond to 200 μm.

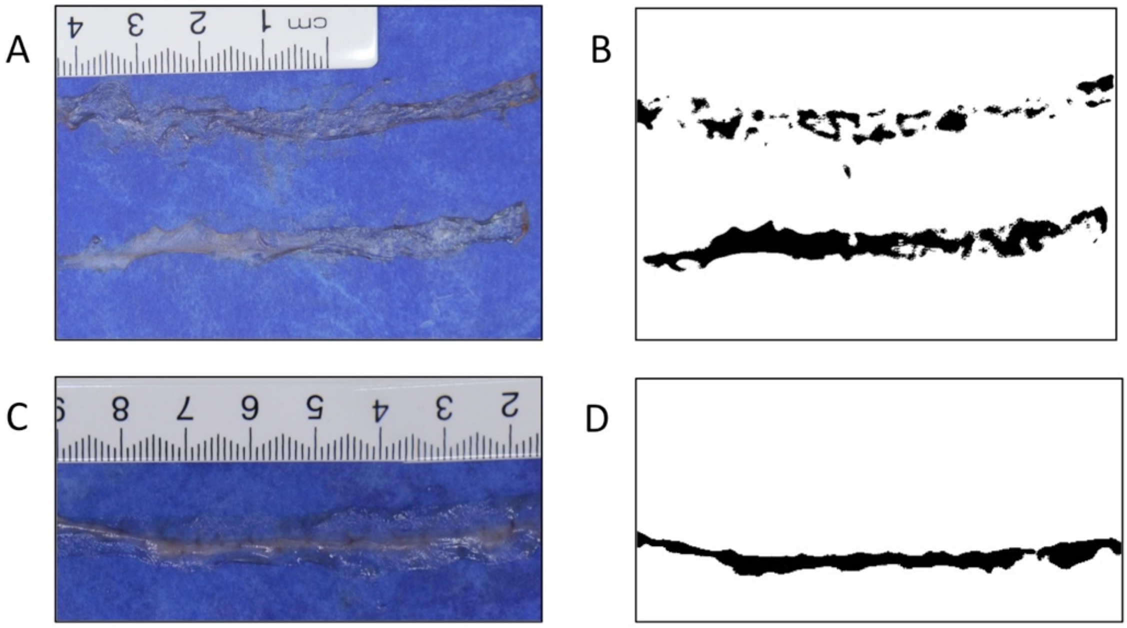

Supplementary Figure 2. The *FeatureCounter* macro can be misled by low quality sample preparations.
Intestinal tissue from APC+/+ (WT) mice was collected and processed as described in the methods.
(**A**) Photograph of curled edges (top section) and dried (bottom section) SI preparations. (**B**) *FeatureCounter* generated tumour masks identify curled edges (top section) and dried areas of SI as interesting features that might be Ad. (**C**) A section of SI with remaining excess fat. (**D**) Corresponding tumour mask showing that excess fat is identified as an interesting feature by *FeatureCounter*.

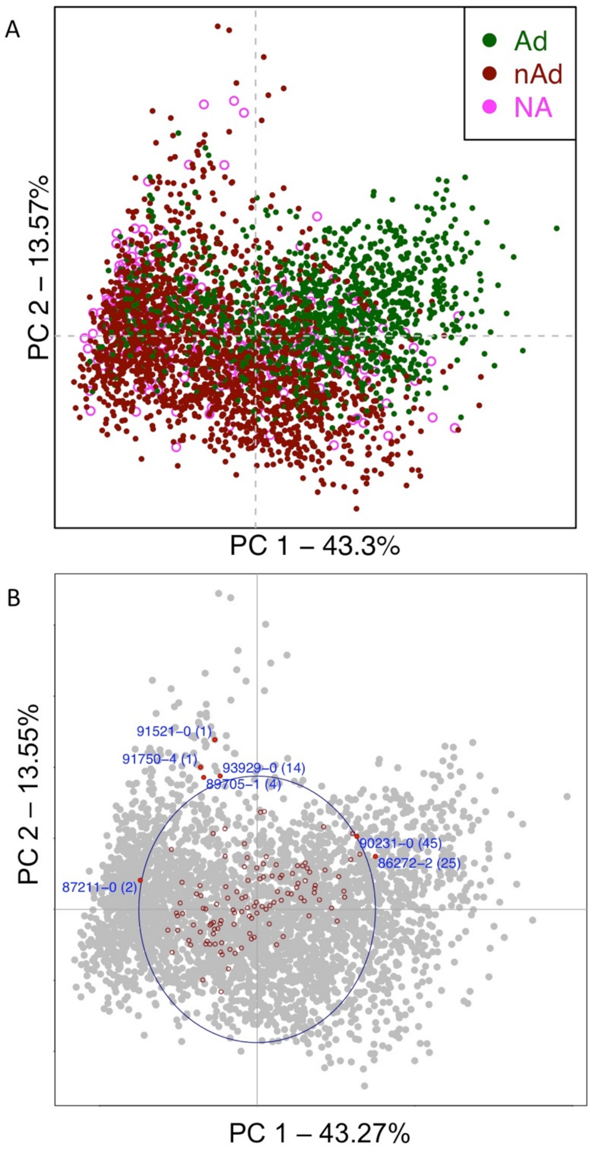

Supplementary Figure 3. Principal Components Analyses of LDA-generated image features.
PCA was applied to features with 22 measures (after transformation and filtering) from 120 mice. (A) PCA applied to 3188 Adenoma (Green) and Non-Adenoma (Red) features. Additionally, 259 Not Assigned (Pink) features are illustratively projected onto the plot.
(B) PCA applied to 3447 (Adenoma, Non-Adenoma and Not Assigned) features from 120 mice. Features are indicated as grey dots. Mouse barycentres are indicated as red circles. Red dots with blue labels indicate mice with distinctly non-central barycentres, i.e. potentially biased mice. Parentheses give feature counts in those mice.

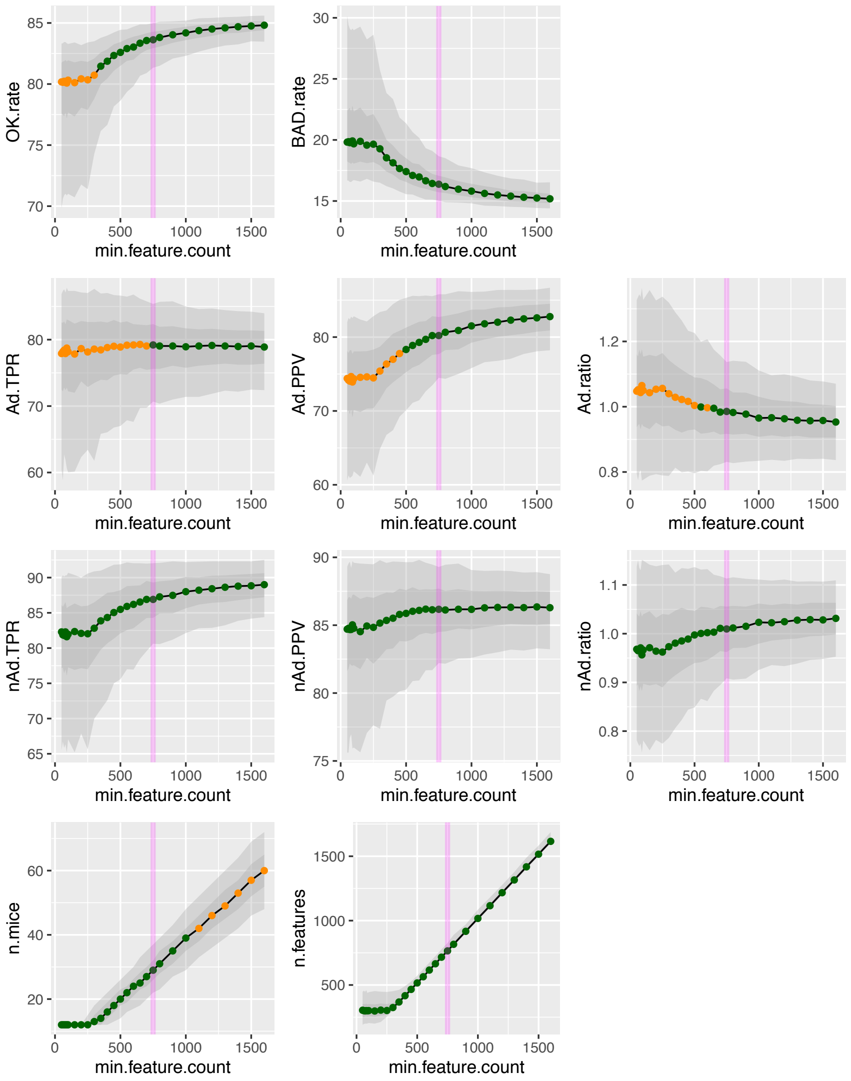

Supplementary Figure 4: Selecting a minimum feature count for bootstrapping sub-sampling datasets.

For several values of minimal feature count (broadly taken between 50 and 1600), performance metrics were calculated across 1000 sub-sampled datasets, built as described in the methods. Each plot represents the 5% - 95% quantiles (light grey area), 25%-75% quantiles (dark grey area), and 50% quantile (i.e. median, black line) for one metric. The dots along the line are coloured according to selection conditions we established (OK.rate 5th percentile ≥ 75% ; Ad.TPR 5th percentile ≥ 70% ; Ad.PPV 5th percentile ≥ 70% ; Ad.ratio between 0.80 and 1.20 ; n.mice 95th percentile ≤ 50) to help guide our selection of a minimal feature count (green: passes condition; orange: fails condition) necessary for appropriate bootstrapping sub-sampling-based validation. The vertical magenta line indicates our final choice for minimum feature count to use in the sub-sampled sets. OK.rate: percentage of correct predictions over total predictions; BAD.rate: complement to OK.rate; Ad.TPR: True Positive Rate on Adenoma predictions; Ad.PPV: Positive Predictive Value on Adenoma predictions; Ad.ratio: ratio of number of predicted Adenomas over actual number of Adenomas; nAd.TPR, nAd.PP, nAd.ratio: same as for Adenomas, applied to not-an-Adenoma features; n.mice, n.features: number of mice & number of total features included in sub-sampled dataset.

Supplementary Table 1: Automatic thresholding and filtering to select “image features” that are potentially adenomas.

Step	Action
1	Min/max range set to 60-200 to exclude background noise
2	Threshold set to 75 (of 255 grey levels)
3	Remove dark outliers of radius 20 with threshold 50
4	Remove light outliers of radius 20 with threshold 50
5	Dilate to increase edge diameters slightly
6	Watershed to split multiple joined features into component parts
7	Analyze Particles with "size=0.2-Infinity"

Supplementary Table 2: ImageJ “analyse particles” variables measured by *FeatureCounter* and their processing.

Measurement	Description	Shift?	Log-transform?	Min.	Max.
Area	Area of feature		Yes		
Perimeter	Perimeter of feature		Yes	0	2
Mean	Mean grey value within feature				
StdDev	Standard deviation of grey value within feature				
Mode	Modal grey value within feature				
Min	Minimum grey value within feature	Yes	Yes		
Max	Maximum grey value within feature				
Median	Median grey value within feature				
Skew	3rd order moment about the mean of grey values in the feature	Yes	Yes	0.25	1
Kurt	4th order moment about the mean of grey values in the feature	Yes	Yes	0	1.5
Major	Major axis of an ellipse fit to the feature		Yes		
Minor	Minor axis of an ellipse fit to the feature		Yes		
Angle	Angle between the axes of the ellipse fit to the feature				
Circularity	Measurement of circularity of the feature				
AR	Aspect ratio of the ellipse fit to the feature - major over minor		Yes		
Round	Roundness - inverse of aspect ratio				
Solidity	Ratio of area to area of a convex hull fit to the feature				
Feret	Segment of longest distance between two points along feature boundary		Yes	-0.3	1.5
FeretAngle	Angle of the Feret segment				
MinFeret	Minimum caliper diameter of the Feret segment		Yes	-0.5	1.5
IntDen	Integrated density: estimated sum of grey values in the feature		Yes		
RawIntDen	Raw Integrated density: sum of grey values in the feature		Yes		

Feature measurements returned by ImageJ's "Analyse Particles" function within *FeatureCounter*, with subsequent transformations and filtering information for use.
C.F. https://imagej.nih.gov/ij/docs/guide/146-30.html (link pulled 12/02/2019) in the Linear Discriminant Analysis.

Supplementary Table 3. LDA indicator quantiles for Adenomas across bootstrapping datasets.

Quantile	Ad.TPR	Ad.PPV	Ad.ratio	Mice	Features
0%	57.62	65.78	0.68	15	750
5%	70.13	73.95	0.82	22	751
25%	75.82	77.84	0.92	26	757
50%	79.12	80.25	0.98	29	766
75%	82.17	82.71	1.05	33	781
95%	85.77	85.76	1.15	37	827
100%	91.01	90.64	1.36	45	959

Quantile: Target quantile (in %) across all 4000 sub-sampled datasets comparing LDA-identified adenomas to the CALL Adenomas. Ad.TPR: True Positive Rate of Adenoma callings; Ad.PPV: Positive Predictive Value of Adenoma callings; Ad.ratio: Ratio of the number of Adenomas called by LDA over the number of True (CALL) Adenomas; Mice, Features: Number of mice and features included in the sub-sampled dataset (with replacement), respectively.

Supplementary Table 4. LDA indicator quantiles for not-Adenomas across bootstrapping datasets.

Quantile	nAd.TPR	nAd.PPV	nAd.ratio	Mice	Features
0%	69.51	75.65	0.76	15	750
5%	79.99	81.99	0.90	22	751
25%	84.39	84.59	0.97	26	757
50%	87.01	86.13	1.01	29	766
75%	89.31	87.64	1.05	33	781
95%	92.14	89.46	1.12	37	827
100%	95.70	92.41	1.22	45	959

Quantile: Target quantile (in %) across all 4000 sub-sampled dataset comparing LDA-identified not-Adenomas to the CALL not-adenomas; nAd.TPR: True Positive Rate of not-Adenoma callings; nAd.PPV: Positive Predictive Value of not- Adenoma callings; nAd.ratio: Ratio of the number of not-Adenomas called by LDA over the number of True Adenomas; Mice, Features: Number of mice and features included in the sub-sampled dataset (with replacement), respectively.

List of materials available from GitLab (https://gitlab.com/gringer/featurecounter/):

MD5 Hash	Path	Description
89aaa67921d2a58181d74c492590c039	./Autoscale.ijm	Macro to add an image scale when it contains a horizontal ruler
56aa13bdc1b4330debcc63a11a297d42	./MeasureFeatures.ijm	Macro to isolate, mark, and calculate statistics for interesting image features
7ac61644fadd7cb6680e9e27c4b52c79	./SubtractBlue.ijm	Macro to subtract the blue background from an image
e3982ca783a403b770382b6c771cebcd	./LICENSE.md	Software license (ISC / free software, non-copyleft)
acaae6bd455642f85e8b205578d2b6ff	./README.md	"Feature Counter -- ImageJ macros to help identify tumour-like areas of an image"
bf5eede59380b4e7f1aef43e53509a4a	./Sample_Photography.pdf	PDF for macro documentation
0498dcadabca6e7906765752861258fc	./Sample_Photography.tex	Text-file source for macro documentation
d536a191a38ac41c9989d85b1fc54dc5	./examples/images/SI81355-1.jpg	Example image: negative, light background
ad2368cc180edeed0627ac570fb2e929	./examples/images/SI90230-3.jpg	Example image: negative, medium background
05da5b15e5b75397265728905861d37d	./examples/images/SI90230-4.jpg	Example image: medium to high adenoma burden, mostly compound features of large size
2c20781eb489003e7420ba1e40e75a89	./examples/images/SI90231-0.jpg	Example image: medium to high adenoma burden, mostly separated features of medium size
8eba38b142e27d7f64983027f42c82c9	./examples/images/SI92332-0.jpg	Example image: low adenoma burden, blurry image, mostly separated features of medium size
a0ebdffcc17cd518c3e1bdbcce3af263	./examples/images/SI92335-0.jpg	Example image: negative, medium background, slanted ruler
a3e974619c14bfdeb315cc3fc26765a8	./examples/images/SI93929-3.jpg	Example image: negative, medium background, chopped off ruler at one side
dace1dcb7634798862f756a6fc17ff50	./examples/images/SI93930-1.jpg	Example image: negative, medium background, high light reflection
b08cbb0fdbabc0025aa122cf069499d3	./examples/images/SI94445-0.jpg	Example image: low adenoma burden, mostly compound features of large size
f9441940c36c28b8d8e55d54886465bf	./examples/images/SI94445-1.jpg	Example image: low adenoma burden, lots of reflection and non-adenoma features
34f298c079ff485840f926ad5efd573e	./examples/images/SI94643-1.jpg	Example image: low adenoma burden, mostly separated features of large size
545a7a3919399950dd5868ccde4e1670	./examples/images/SI95080-2.jpg	Example image: low adenoma burden, mostly separated features of large size
14c46dbb85db82ab197dbc02ab48cd4a	./examples/images/SI95779-2.jpg	Example image: low adenoma burden, mostly separated features of large size
63bd6755a83698122130e547fddd6086	./examples/SimpleLDA.R	Example R code to carry out an LDA on feature statistics
3fad2526eebbdb2d3f9a5c63800d043c	./examples/FeatureData.	Example input for LDA code
32a178fe39769c994e0fa796e26e676c	./examples/lda_output.txt	Example output of LDA code
d1d1c5215c8341b77a45fcebf146fe18	./examples/Data_Apc_Min.xls	Example spreadsheet containing aggregate mouse-level metadata and image feature data
021ebeeda7ceefdd5de284296679a8ba	./illustrations/small_R-B_DkrPaint.jpg	Documentation illustration: after removing blue background
8ba9d0139bb015e438d92f8c04e6b74b	./illustrations/photography_setup.svg	Documentation illustration: camera setup for good image photography
5fa337f208fbece1435ac204ceec1281	./illustrations/photography_setup.pdf	Documentation illustration: camera setup for good image photography (PDF version)
89ef9ae1f234389a272d668f5eb2d513	./illustrations/stitching_setup.svg	Documentation illustration: image / photo layout for good stitching
c7927127856807b19088cb15c23147b6	./illustrations/stitching_setup.pdf	Documentation illustration: image / photo layout for good stitching (PDF version)
d8629e5eb538626b3041219d84d2ef4f	./illustrations/small_foundRegions_DkrPaint.jpg	Documentation illustration: identified regions overlaid onto the original image
57d06ec19f5b16bd69c2989242fdd493	./illustrations/small_DkrPaint.jpg	Documentation illustration: original image with highlighted ruler scale points

Tumour images are available from Zenodo repository doi:10.5281/zenodo.3365777.
